# Moral challenges and understanding of clinical ethics in Tanzanian hospitals: Perspectives of healthcare professionals

**DOI:** 10.1111/dewb.12467

**Published:** 2024-10-19

**Authors:** Shija Kevin Kuhumba, Bert (A.C) Molewijk, Jan Helge Solbakk, Nandera Ernest Mhando, Trygve Johannes Lereim Sævareid

**Keywords:** clinical ethics support, clinical practice, ethics, ethics education, healthcare professionals, moral challenges, Tanzania

## Abstract

Healthcare professionals encounter many moral challenges in their daily clinical practice. However, there have been few studies on the subject matter in Tanzania. This study aims to provide an account of moral challenges faced by healthcare professionals in Tanzanian hospitals, their understanding of clinical ethics, and the ethics education they have received. Many participants reported receiving some kind of ethics training through formal education and on‐the‐job training. Some participants understood ethics in healthcare settings as adherence to established laws, regulations, guidelines, procedures, norms, and rules essential in clinical practice. Analysis of the data identified four themes of moral challenges. These challenges are related to 1) decision‐making and communication in clinical practice, 2) scarcity of medical resources and prioritization in clinical practice, 3) withdrawal of curative treatment, and 4) conflicts between professional judgment, religious convictions and adherence to alternative treatments. Based on the findings, we suggest a context‐sensitive form of clinical ethics training to prepare healthcare professionals to recognize and address these moral challenges.

## 1 INTRODUCTION

Healthcare professionals encounter multiple moral challenges in their daily clinical practice.^1^ A moral challenge in healthcare can occur when important decisions have to be made, in cases of moral uncertainty about what should be done, in case of a disagreement about decisions, or a conflict between ethical principles and values. It can be associated with moral questions such as who should decide over a patient's life and death, when should the patient's autonomy not be respected, how much power should next of kin have over a patient's care, and when is it right to withhold or disclose the truth to the patient and next of kin.^2^ Moral disagreement considers situations in clinical settings where healthcare professionals, next of kin, and patients disagree about care and related moral values.^3^ Several international studies^4^^,^ 5 ^,^ 6 have identified several moral challenges encountered by healthcare professionals, including lack of resources;^7^^,^ 8 disagreements among healthcare providers;^9^^,^ 10 challenges related to informed consent processes;^11^^,^ 12 challenges associated with disagreement among patients' family members^13^; issues related to disclosing medical information and maintaining confidentiality^14^; encounters between different cultures in the delivery of healthcare services^15^; decision‐making and care at the end of life.^16^ However, all these studies report on moral challenges healthcare professionals face in high‐income countries.

Due to differences in ethical and cultural traditions, there are reasons to believe that moral challenges in healthcare settings in the Sub‐Saharan African context might differ. For example, in the Sub‐Saharan African context, more emphasis is placed on the community‐based approach to moral challenges in healthcare settings. The community‐based approach is grounded on indigenous values salient in the sub‐Saharan region, according to which an action is right just insofar as it expresses respect for communal or, equivalently, friendly or (broadly) loving relationships ones in which people both identify with each other (share a way of life) and exhibit solidarity with each other (care for others' quality of life).^17^ This conception might differ from the individualistic approach in the Western and North American context where the four principles framework of Beachamp and Childress is dominant. The principles of respect for autonomy, beneficence, non‐maleficence, and justice are usually interpreted in individualist ways, by referring to a given person acted upon.^18^ For instance, the principle of respect for autonomy fails to acknowledge the fundamental importance of understanding persons within the nexus of their communal relationships.^19^ Generally, in the Sub‐Saharan African worldview community is prized and individuals are bound up with their communities. Decisions about one's body and life are, therefore, not necessarily taken by individuals acting alone, but in engagement with their families and communities.^20^ Studies that have attempted to explore moral challenges and dilemmas in healthcare settings in Sub‐Saharan Africa report cases that are either directly or indirectly connected to community and family settings.^21^^,^ 22 ^,^ 23 ^,^ 24 The reported challenges are related to the scarcity of medical resources and the patient‐family's economic conditions;^25^^,^ 26 disclosure of information and issues of confidentiality^27^; moral challenges associated with abortion and termination of pregnancy;^28^^,^ 29 challenges concerning cultural issues and disagreement with family members^30^; challenges related to lack of collaboration between physicians and nurses in healthcare services^31^; end‐of‐life issues^32^; traditional treatment versus alternative medicine^33^; and challenges related to use of coercion in treatment of patients.^34^ Most studies conducted in Tanzania^35^^,^ 36 ^,^ 37 focus on particular moral challenges encountered by healthcare professionals working with patients with a specific disease. They include communication and truth‐telling,^38^ scarcity of resources in healthcare settings,^39^ as well as family disagreement and decision‐making in clinical care.^40^ Therefore, little has been studied regarding moral challenges in healthcare delivery in the broader context of Tanzanian healthcare settings.

Addressing moral challenges in clinical practices requires moral competence, including the ability to identify a moral problem, knowledge about moral and socio‐cultural aspects of care, reflection on a moral issue and possible alternatives of action, and the ability to make wise choices and carefully manage morally challenging situations.^41^ Moral competence is important in enhancing healthcare professionals' ability to respect patients' rights and provide quality care.^42^ There are various ways to develop the capability of healthcare professionals' moral competency to identify and solve moral issues in clinical practice.^43^ Ethics education is considered one of the ways to develop such a competency.^44^^,^ 45 However, some studies conducted in Africa report limited ethics training among healthcare professionals due to a lack of trained ethicists, and clinical ethicists in particular, as well as little emphasis on clinical ethics.^46^

Ethics education would enhance moral competency and understanding of ethics in clinical practice. As a result, understanding of ethics or clinical ethics could enhance healthcare professionals' capacity to recognise and identify moral issues in clinical practice. Some studies have reported the relevance of clinical ethics support in enhancing the moral and professional competency of healthcare professionals.^47^^,^ 48 However, there are a few studies conducted in Sub‐Saharan Africa on how healthcare professionals understand ethics or clinical ethics. For instance, physician residents based in Egypt expressed an understanding of bioethical principles. Most residents mentioned at least 1 of the 4 patients' rights: privacy, obtaining informed consent, confidentiality, and beneficence.^49^ A quantitative study conducted in Ghana found that 77.7% of the nurses reported good knowledge of ethical standards in the nursing profession, 66.6% reported moderate knowledge, and 5.7% reported low knowledge.^50^

The current study is part of the ETHIMED (Enhancing Ethics and Integrity in Medical Research and Clinical Practice) project. ETHIMED project is a collaborative venture between the University of Oslo through Centre for Medical Ethics, the University of Dar es Salaam through the Department of Philosophy and Religious Studies, and the University of Rwanda through the College of Medicine and Health Sciences. In Tanzania, the ETHIMED project focuses on capacity building of clinical ethics for healthcare professionals, conducting research on clinical ethical issues, and establishing the first clinical ethics committee in Tanzania.

The study findings contributed to the preparation for the implementing project activities including clinical ethics training. In addition, the preliminary findings of this study informed the establishment of the first clinical ethics committee at one of the study sites in February 2024. Therefore, this study aimed at exploring moral challenges faced by healthcare professionals in Tanzanian hospitals, awareness of ethics and clinical ethics, and their received ethics education among participants.

## 2 METHODS

A qualitative approach to data collection and analysis was selected. We used the standards for reporting qualitative research (SRQR) as the checklist for reporting qualitative studies.^51^ Participants were recruited from three tertiary hospitals. These hospitals were purposively selected because they serve many patients with diversified backgrounds. Also, hospitals were purposely selected because they serve patients with more complex and severe cases. In their capacity as teaching hospitals, the selected study sites are affiliated with institutes of health and allied sciences, where medical students, nurses, resident physicians, and other healthcare professionals receive their training. The study participants were recruited from intensive care units, and emergency and internal medicine departments. Although moral challenges will probably be present at any clinical department in Tanzania, we needed to make a selection to limit the amount of data and contexts. Therefore, prior to conducting fieldwork, the first author engaged in preliminary discussions with various Tanzanian healthcare professionals to identify specific departments that are more likely to encounter moral challenges. Through these discussions, three specific departments were identified. The rationale for consulting these healthcare professionals was that none of the authors had working experience in Tanzanian healthcare settings. In addition, physicians and nurses from the selected departments with more than three years of experience participated in the study.

### 2.1 Participants and study settings

The first author contacted the selected departments and inquired that the heads of departments provide access to participants who met the inclusion criteria. Then, participants were recruited and contacted directly by the first author to seek an appointment for the interview. A total of 36 participants were recruited, whereby females were 19 (52%) and 17 males (48%). Among the participants were 15 physicians (41.7%) and 21 nurses (58.3). The study included 16 participants from hospital A, and 10 participants from hospital B and hospital C. (see Appendix 1).

**Figure 1:**
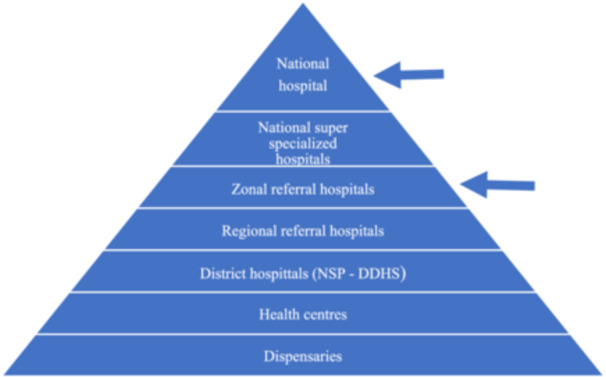
Structure of the healthcare system in Tanzania.

In this study, it is crucial to provide a brief description of the Tanzanian healthcare system so as to capture the broader environment in which the study is situated. Tanzania is a low‐middle‐income country in Sub‐Saharan Africa with a population of 61.8 million. The public health care system in Tanzania is organized as a pyramid (Figure 1). At the bottom of the pyramid are located dispensaries which offer basic outpatient care. Dispensaries are staffed by clinical assistants and enrolled nurses and are followed by health centres, staffed by clinical officers and nurses, offering more comprehensive care. District hospitals provide both outpatient and inpatient services. In areas without public hospitals, non‐state providers (NSPs) fill the gap, including private‐for‐profit providers, and private‐not‐for‐profit providers such as faith‐based organizations (FBOs).^52^ NSP‐run hospitals are known as designated district hospitals (DDHs), and are subsidized by the government. Multiple districts are grouped into regions, each with a regional hospital. Zonal referral hospitals take care of multiple regions categorized into zones. Finally, at the top of the pyramid are super specialized and national hospitals.

Tanzanian health sector funding comes from two main sources: central support financed by the government of Tanzania's general tax revenue and development partners (DPs) support. DPs provide pooled funding both through general budget support (GBS) and the Health Basket Fund (HBF), a form of sector budget support.^53^ Our study participants were recruited from the national hospital and zonal referral hospitals. (see Figure 1).

### 2.2 Data collection

The interview guide (see Appendix 2) was collaboratively developed by the first, second, fourth, and last authors. This collaboration ensured that the guide was comprehensive and aligned with the study's research questions, rather than reflecting on a single perspective. The interview guide developed for this study ensured structured and consistent data collection, thereby mitigating potential researcher bias in several ways. First, it minimized the likelihood of the researcher engaging in unintentional conversations that might reflect personal perspectives and interests. Second, it established a framework that reduced the researcher's influence on responses that could align with their expectations. Third, it maintained a focused and consistent examination of the core themes of the study, thereby offering a basis for systematic comparison of the collected data.

The first author interviewed the participants at their workplace. Semi‐structured interviews were conducted between June and September 2023 and lasted, on average, 40 minutes. All interviews were conducted in the Swahili language. Upon obtaining informed consent, the first author recorded interviews, transcribed the audio‐recorded interviews verbatim and translated them from Swahili to English.

### 2.3 Data analysis

Reflexive thematic analysis (TA) was employed to analyze the collected data. The reflexive TA is a qualitative analysis framework developed by Braun and Clarke.^54^^,^ 55 ^,^ 56 Reflexive TA is a method for identifying, analyzing, and reporting patterns (themes) within data.^57^ The iterative inductive data analysis entailed (i) familiarization with the data, (ii) generation of initial codes, (iii) search for themes, (iv) review of themes, (v) definition and naming of themes, and (vi) drafting the report. At this phase, data extracts were incorporated into the result section of the article.

After transcribing the data, the first author read the data sets repeatedly to get familiarized with the data. Then, the first author developed a summary of the key findings, and other authors commented. The first author created initial codes after familiarizing with the data, and the other authors reviewed and provided feedback on these codes. Also, the first author developed themes by collecting the initial created codes. The developed themes were thoroughly reviewed by last and second authors. Then, the developed themes were defined and named. Finally, the first author wrote a preliminary report, and other authors commented and added constructive inputs.

In the analysis, we used NVivo 12 software in data coding and identifying recurring themes and subthemes. The interview transcripts were imported into the NVivo 12 software. Finally, the first author developed a table (see Appendix 3) indicating themes, sub‐themes, codes, and illustrative quotes, which was reviewed by other authors.

We recognize that our analysis is shaped by our professional and academic backgrounds, skills, experiences, and social positioning as researchers.^58^^,^ 59 Authors have diversified academic and professional backgrounds. Two authors have academic training and experience in nursing. One of the authors is trained as a physician, philosopher and theologian. Another author is a social anthropologist, and the first author has an academic background in philosophy and ethics. The diversified academic background among authors has been significant in analysing data, developing themes and developing the paper from multidisciplinary perspectives. In terms of social positioning, three authors are from Europe, while two are from Tanzania. However, three authors from Europe have experience in ethics training from intercultural perspectives. In addition, three authors have been involved in ethics training and establishing the first clinical ethics committee in Tanzania. Due to intercultural experiences, data findings were interpreted from intercultural perspectives on moral issues in clinical practices.

### 2.4 Ethics consideration

Ethical approval was obtained from the National Health Research Ethics Committee (NATHREC) in Tanzania under the National Institute for Medical Research (NIMR), relevant regional research ethics committees, Independent Review Boards (IRB) at the selected hospitals, and the University of Dar es Salaam Research Ethics Committee (UDSM‐REC), as well as the Norwegian Data Protection Services for research (Sikt). Informed consent was obtained from all participants, after providing detailed information about the study both orally and in writing. As the interviews were concerned with sensitive topics, in this paper we included limited demographic information about individual participants to protect their anonymity. Our dataset has more demographic information.

## 3 RESULTS

In this section, we present healthcare professionals' ethics education, their perspectives regarding understanding of ethics/clinical ethics, and the moral challenges they encountered in clinical practice.

### 3.1 Ethics education in healthcare

Almost all participants acknowledged having received little training in ethics in general while attending medical and nursing schools. Two participants were holders of Masters of Bioethics degree. A few had received ethics training either through online courses or workshops. One of the male physicians noted that “*I have been exposed to medical ethics mostly through my initiative to study online”* (*#15, hospital A*).

A small number of nurses had attended a nursing ethics course that primarily focused on professional codes of conduct guiding nursing practice. One of the male nurses said:

…. We were taught how to work efficiently and address customer challenges when providing healthcare services…. (#20, male nurse, hospital B).

Most physicians had medical ethics as a single module under the Ethics and Professionalism course at the medical school. They asserted that the focus was mainly put on professional codes of conduct and instructions on how to follow the codes of conduct for health professionalism. One of the male physicians said: “*I have … received some training on …. the healthcare code of conduct with ethical components* ….” (#*10, male physician, hospital A)*.

On the ethics and health professionalism course, it was noted that very little consideration was given to ethical reflections due to a lack of trained ethicists and bioethicists:

… the focus was on professionalism and the patient‐doctor relationship, but the actual ethical aspects, such as principles, dilemmas, and decision‐making, were only touched upon very briefly…. Unfortunately, when we were studying, no trained ethicists were available, so experts from the medical profession often taught us. They put more focus on the medical science aspects rather than ethics. (#14, female physician, hospital A).

Some participants indicated that the time allocated for medical ethics in the curriculum was perceived as insufficient. One physician emphasized the need to develop a comprehensive ethics course to aid physicians in handling an increasing number of complex moral issues (#16, female physician, hospital A).

### 3.2 Understanding of ethics and clinical ethics

The study findings indicate that participants had varying understandings of ethics and clinical ethics. Some participants regarded ethics as a synonym of clinical ethics. When asked to give their views on how they understand ethics in general and clinical ethics in particular, many participants took time before answering, and some asked for clarification about the question.

Some participants expressed an understanding of ethics in connection with principles. Respect was one such example, and it entailed ensuring that “patient's rights are upheld without causing harm” (*#11, female physician, hospital A*). Other examples were “doing something beneficial for the patient and maintaining the confidentiality of the information provided by the patient” (*#6, male physician, hospital A*). Another participant reported that dignity could be achieved through the way services “are provided, the reception and management of patient or client information” (#25, *male nurse, hospital B*).

Ethics and clinical ethics were understood by others as adherence to established laws through “meeting their needs and offering quality services” (*#32, female nurse, hospital C*), and “following regulations, guidelines, and procedures” (*#33*, *male physician, hospital C*) guiding clinical practices. Fulfilling and abiding by the duties as an employee was viewed as upholding the ethics of the job. In addition, some study participants defined ethics in connection with the norms and rules that are essential in clinical practice. Ethics in clinical practice was described this way: “*…. it could mean rules established to guide healthcare professionals in providing healthcare services…*” *(#1, male nurse, hospital A)*.

Moreover, ethics was viewed by some as norms that are essential in the clinical practice related to communication because kindness and cheerfulness could be given to patients through it. Values such as honesty and exhibiting good customer care to the patients in the clinical practice were also associated with clinical ethics.

### 3.3 Moral challenges in clinical practice

Four themes of moral challenges were developed from the analysis. We noted that when asked to give their view on moral challenges in clinical practice, many participants hesitated and took time before answering. The first author explained the meaning of moral challenges and gave a few examples. Most of the participants had themselves experienced morally challenging situations. Only a few participants reported having not encountered such situations. Instead, they were aware of moral challenges experienced by colleagues in their department.

#### 3.3.1 Theme 1. Scarcity of medical resources and prioritization in clinical practice

Insufficient medical resources created moral challenges in clinical practice. First, limited medical resources hinder healthcare personnel from delivering adequate healthcare services to all patients in need; hence, they are left with the dilemma of choosing which patients to prioritize with the risk of neglecting others.

… there are situations where no matter what we do, a child cannot recover because they were born with multiple disabilities or … their brain has been damaged due to lack of oxygen. Babies under such health conditions require full‐time ventilation for their survival. But the ventilation machines are limited. Then another child with better conditions comes along who needs a ventilation machine. Now you are faced with a dilemma: the law prohibits taking off the machine a baby with multiple disabilities. Even if the child survives, his quality of life may not be good. So, do you turn off the machine, or wait until the terminally ill child dies? (#1, male nurse, hospital A).

I faced a case where there were three patients needing blood, but the laboratory only had one unit of blood from a group O donor, which could be given to only one of them. (#26, female physician, hospital B).

Second, some participants pointed out how limited medical resources hindered them from fulfilling their obligation of treating patients during the COVID‐19 pandemic:

There was insufficient protective equipment, so there were moments when you would see a patient suffering but couldn't get close. (#25, male nurse, hospital B).

#### 3.3.2 Theme 2. Decision‐making and communication in clinical practice

Challenges related to decision‐making could occur as part of collaboration between family members, patients, and the team of healthcare professionals. One participant noted, “*In our setting, very often an entire family is significant in deciding a patient's treatment because we have a communal approach*” (*#9, female nurse, hospital A*). Furthermore, there are moral concerns about patient privacy and confidentiality when family members, friends, or neighbours handle patient's information:

Patients don't make decisions for themselves. Even their confidential information is handled by their family members, friends, or neighbours…. (#9, female nurse, hospital A).

Sometimes family members decide to discharge the patient for financial reasons:

We had a patient in the ICU who was on maximum life support, and the relatives came and requested to take the patient home because they couldn't afford to care for them here…. (#34, female physician, hospital C).

…a sick child who needed close medical attention and care from a doctor, but the parents wanted the child to be taken off oxygen and taken home. …. they went home against medical advice … it seems the mother herself wasn't ready to see her baby discharged against medical advice. Even though her decisions might differ from the husband's, he is still the one with the means and the provider of finances for medical treatment (#11, female physician, hospital A).

In other instances, participants claimed that neither patients nor family members were involved in making decisions about their treatment. One of the participants asserted that:

A patient undergoes a procedure, for example, intubated, and is on a ventilation machine. There is no process for them to be part of the decision‐making, even though they are informed about the situation. (#9, female nurse, hospital A).

Participants expressed that complex cases may create moral challenges when the decision‐making process involves a team of healthcare professionals. One of the participants shared a situation involving a pregnant mother who was brain dead but still had a heartbeat. The challenge for the healthcare professionals was deciding between allowing the pregnant mother to die with her unborn baby or whether the unborn baby should be delivered prematurely. One of the male nurses claimed that:

We advised them to deliver the baby quickly because the mother wouldn't recover because she was seven months pregnant, the baby could have survived because babies born at that stage can survive. But there were discussions about what would happen if the baby was delivered prematurely… They presented their (clinicians’/healthcare professionals') arguments, but the challenge was not resolved… eventually, the pregnant woman's heart stopped before delivering the baby. (#25, male nurse, hospital B).

Involvement of members of the extended family in deciding on organ donation could create communicative and advisory difficulties:

…a young man had decided to donate a kidney to his uncle, but in front of people, his mother had accepted, and then later, when they were alone, she told him not to come back home if he donates his kidney. So, I was in a difficult situation to advise a potential donor whether to donate the kidney or accept the ‘ ‘mother's position. (#14, female physician, hospital A).

Communication of bad news to patients and relatives was identified as a moral challenge. One of the participants acknowledged that it was challenging to disclose bad news to the patient's family members while the patient's heart was still beating:

For example, there are moments when a patient comes with severe illness, but you find they are having brain death. Do you remove them from the machine? How do you tell the parents or family members? How do you convince them when they see the heartbeat of their patient, yet in the actual sense, it is the machine helping the patient to breathe? (#15, male physician, hospital A).

#### 3.3.3 Theme 3. Withdrawal of treatment

Uncertainty of when to minimize or stop aggressive treatment was challenging. A perceived lack of or limitations of guidelines and regulations could exacerbate this.

According to guidelines, you can't remove them from life support machines until they die naturally. However, keeping them on life support increases the medical costs for their families, who sometimes, after understanding the patient's condition, request to have them removed from life support to reduce the financial burden. (#19, male nurse, hospital B).

… when you have a patient who won't recover and is on oxygen, and then another patient needs a machine to save their life potentially. However, Tanzanian guidelines don't allow us to remove the ventilation machine from the terminally ill patient, even if it could save the life of a patient with a high chance of recovering. (#18, female physician, hospital B).

Family members may prepare to sell their physical property to cover medical treatment for a critically ill‐patient. In such situations, healthcare professionals encounter the challenge of advising family members whether to withdraw treatment or minimize care.

they may require dialysis, which is very costly, and at the same time, the patient's condition may have deteriorated significantly… (#19, male nurse, hospital B).

#### 3.3.4 Theme 4. Conflicts between professional judgment, religious convictions and alternative treatments

Religious convictions or alternative medicine could trigger patients and family members to decline medical treatment. One example was executing the termination of pregnancy as a medical intervention, which conflicted with the family's religious beliefs:

I have faced the challenge of a pregnant woman with a serious health issue that required the termination of her pregnancy to save her life. Given her condition and the stage of her pregnancy, termination was necessary. However, her family and relatives believed that God would perform a miracle, so they insisted on keeping the pregnancy. (#20, male nurse hospital B).

Some participants highlighted situations whereby believers such as Jehovah's Witnesses rejected treatment that required blood transfusion. The participants reported cases they encountered where the refusal of a potentially life‐saving blood transfusion seemed a controversial choice that challenged their professional judgment to act in the patient's best interests:

a young boy, around 8 years old … His problem was sickle cell disease, and his blood level was deficient. … They told us that if we gave him blood, we would be sued…. (#36, male nurse, hospital C).

Some patients and family members declined treatment in preference to alternative treatment. In some instances, alternative treatment was considered as a therapy for some diseases that are associated with supernatural beliefs. Some participants noted that:

It was a young person who had been in an accident and got a fracture on one of his legs. He also had an open wound that was bleeding. Their family members refused to allow him to undergo surgery because they preferred alternative treatment (traditional medicine) …. (#28, female nurse, hospital C).

… the patient left the hospital premises and went for spiritual therapies – prayers, but unfortunately, the patient died. (#12, male nurse, hospital A)

## 4 DISCUSSION

In this Discussion section, we are reflecting upon the study findings and comparing them with previous international studies on the subject matter.

### 4.1 Ethics education

A considerable number of participants reported to have been trained in ethics as deductively applying or following moral or prescriptive rules or norms. Most of the participants were not trained to reflect themselves on how to handle and balance the various moral challenges occurring in clinical practice. To strengthen clinical ethics in Sub‐Saharan Africa, and Tanzania in particular, we believe that capacity building projects are valuable in strengthening competence, understanding, and training in recognizing and handling moral challenges within clinical ethics. Capacity building projects with international mutual exchange might be beneficial for all partners, broadening the perspectives and competence of those involved.

Implementation of capacity building projects should consider conducting empirical research on the local context; having normative discussions on locally experienced moral challenges; and developing and implementing education and ethical support systems.^60^ Moreover, capacity building projects should be developed locally in close collaboration with experienced (clinical) ethicists and healthcare professionals concerning specific dilemmas perceived as relevant and context‐based.^61^ This calls for mutual collaboration between all partners in co‐designing capacity building programmes that capture the key needs of the local participants. In addition, mutual collaboration could facilitate the integration of contextual values and principles into clinical ethical issues. For instance, Andoh discusses two different conceptions of medical ethics in Africa, namely ethno‐ethics, and professional or academic medical ethics. Ethno‐ethics refers to sets of moral principles rooted in culture and revolves around harmonious coexistence with the cosmos and the promotion, and protection of human life.^62^ Andoh's overall objective is to incorporate African views and approaches to current medical ethics/clinical ethics debates.

Mutual engagement of various stakeholders is essential in integrating African views and perspectives in clinical ethics/medical ethics debates. Stakeholders could include healthcare professionals based in Africa, healthcare training institutions in Africa, bioethics/clinical ethics networks, development partners, bioethicists/clinical ethicists and philosophers, families, patients, religious leaders, and community representatives.^63^ On the one hand, healthcare professionals could present lived experiences of morally conflicting situations in their context. On the other hand, healthcare training institutions could integrate context‐based aspects into clinical ethical issues.

The partners in the ETHIMED project adopted the strategy of co‐developing workshops on clinical ethics by involving experienced experts in clinical ethics support and local healthcare professionals and ethicists.^64^^,^ 65 The workshops aimed at building the capacity of healthcare professionals and support staff on clinical ethical issues such as priority setting in healthcare, ethical reflections on beginning‐of‐life and end‐of‐life issues, and ethical reflections on the use of coercion in healthcare. The collaboration enabled the facilitators to contextualize clinical ethical issues in the Tanzanian healthcare context.

### 4.2 Understanding of ethics and clinical ethics

A large number of participants understood ethics and clinical ethics as adherence to established regulations, guidelines, laws, and procedures guiding clinical practices. A possible explanation for this is how ethics education in health and allied sciences institutions focuses mainly on health professionalism only.^66^ Healthcare professionalism guidelines and norms often deductively dictate the behaviour and decisions of healthcare professionals in their clinical practice. A similar finding has been reported in a study among physicians in Ethiopia.^67^

This understanding of ethics, aligns with professional morality which focuses on standards of conduct that are generally acknowledged and encouraged by those in the profession.^68^ In Tanzania, regulatory bodies such as the Medical Council of Tanganyika (MCT) for physicians and allied sciences professionals, alongside the Tanzania Nursing and Midwifery Council (TNMC) for nurses and midwives, put indeed much emphasis on the enforcement of professional codes of conduct in protecting patients' welfare. This emphasis is also notably evident through institutional guidelines and departmental standard operating procedures that delineate clinical practice parameters.

However, understanding ethics as *only* adherence to professional standards and guidelines tends to ignore ethics as a personal reflection on actions, and ways to jointly deliberate on morally distressing situations in healthcare settings. For instance, the code of conduct for health professionalism has less to say about pertinent ethical issues in healthcare settings such as priority setting in clinical practice in instances of inadequate medical resources, dealing with situations of withdrawing and withholding treatment under some conditions, as well ways to handle conflicting values between patients, family members, and healthcare professionals. We suggest further investigations in the future to understand the contribution and usefulness of introducing clinical ethics programmes and ethics education among healthcare professionals in healthcare settings.

### 4.3 Moral challenges in healthcare settings

#### 4.3.1 Scarcity of medical resources and prioritization in clinical practice

Like in this study, a study based in Tanzania found that resource constraints of medical supplies may leave healthcare practitioners with what was described as unresolvable moral dilemmas.^69^ The scarcity of resources leaves healthcare professionals in morally challenging situations since they are sometimes forced to provide suboptimal care or at times, no care at all to patients.^70^^,^ 71 ^,^ 72 In other instances, healthcare professionals encounter patients with competing needs amid insufficient resources to cater to all patients.^73^

Consequently, healthcare professionals encounter moral distress caused by limited medical resources forcing them to provide suboptimal care in some situations. Moral distress occurs when professionals cannot carry out what they believe to be ethically appropriate actions because of constraints or barriers.^74^ Therefore, clinical ethics support could help healthcare professionals to deal with unescapable forms of moral distress by building moral resilience. Rushton introduces the concept of moral resilience as “the capacity of an individual to sustain or restore their integrity in response to moral adversity”.^75^^,^ 76

Moral resilience considers qualities like personal and relational integrity, buoyancy, self‐regulation and awareness, moral efficacy, and self‐stewardship, which are argued to be needed to respond to complex ethical issues that arise in clinical practice.^77^ Several approaches have been suggested to reduce moral distress through fostering moral resilience among healthcare professionals including the use of ethics consultation,^78^^,^ 79 education and training programmes for healthcare professionals on moral resilience,^80^^,^ 81 moral distress reflective debriefs interventions,^82^ and moral case deliberation.^83^

In addition, scarcity of resources affects patients and family members who need a planned medical treatment, but they are not in a position to afford it. In Tanzania, out‐of‐pocket payment (OOP) accounts for about 22% of the total health expenditure, while health insurance schemes (premium payment) account for about 8%.^84^ OOP continues to be the main means of healthcare financing, thus exposing many people to catastrophic health expenditures.^85^

We recommend the implementation of clinical ethics support (CES) services in healthcare settings. CES are services that aim to support health systems, health care professionals (including ancillary staff, managers and directors), patients and their families when confronted with an ethical concern, question or dilemma.^86^ Delivery of CES services is often categorised broadly speaking into three different models: clinical ethics committees (CEC), individual ethics consultants (EC), and facilitation of moral case deliberation (MCD).^87^ If implemented in healthcare settings, CES could focus on offering on‐job training on moral resilience traits that are necessary in dealing with moral challenges related to limited medical resources and prioritization in healthcare settings.

Besides offering clinical ethics education and moral competence capacity building for healthcare professionals in order to strengthen how they address clinical moral issues and moral resilience, CES services have additional roles in healthcare settings.^88^ First, contributions to the development of institutional guidelines and policies on clinical ethics‐related matters such as priority setting.^89^^,^ 90 Second, providing ethics support at the level of the health system as opposed to the level of patient care.^91^ This role usually entails working through ethical issues involved in areas such as healthcare management, resource allocation, and quality improvement. For instance, in healthcare settings where CES services exist, clinical ethics committees may play an important role in handling issues related to priority setting, including advising and raising awareness of the ethical aspects of resource allocations and promoting fair resource allocation.^92^ Thus, the implementation of CES services could facilitate the drafting of policy guidelines either at the hospital level or department level that address moral challenges in clinical settings such as bedside rationing due to scarcity of medical resources.

#### 4.3.2 Decision‐making and communication in clinical practice

In our study, we found that the community carry a greater weight in African value systems than individuals.^93^^,^ 94 ^,^ 95 For instance, it is always difficult not to inform relatives of the patient's condition, who, in the first instance, are likely to bring the patient to the hospital and, who most of the time, are responsible for the cost of treatment. Thus, a relational approach rooted in Ubuntu ethics has been proposed for decision‐making.^96^^,^ 97

Ubuntu is a fusion of normative ideas that largely inform beliefs, values, attitudes, and practices in Sub‐Saharan Africa. These values are based on ethical beliefs, moral judgments, and ideas, such as prizing communal relationships prevalent among people in the communities, rather than an individualistic approach to respect for autonomy.^98^^,^ 99 Thaddeus Metz suggests that discussions around the nature of communal relations in Ubuntu, depict two relational themes namely ‐ identifying with others, and exhibiting solidarity towards others.^100^ In another study, he argues that a sense of identity and solidarity is characterized by friendship or friendliness.^101^ On one hand, identifying with others entails various actions including thinking oneself as a we (cognition), developing a sense of togetherness or expressing shame or pride in what a group does (emotions), engaging in joint projects (conation), adopting goals consistent with others (volition), as well as coordinating behaviour to realize shared ends (motivation). On the other hand, exhibiting solidarity implies engaging in mutual aid, caring for the conditions of others, exhibiting positive emotions or motives towards others, helping others for altruistic reasons and acting for the sake of one another.^102^

Based on our findings about the moral challenge of disclosing a patient's confidential information about his/her health condition, it is pertinent to ask whether it is morally justifiable for a healthcare professional to disclose family members who incur medical costs and have due responsibility for the patient's wellbeing. On Ubuntu grounds,^103^ various persons suggest that there are reasons to justify that healthcare professionals are not obligated to maintain patient confidentiality in the way that is currently expected in the West.^104^ Patient's relationship with family might mean that the family is entitled to know about patient's health.^105^ For example, regarding this view, Murove states:

Communal participation in the individual's illness, and treatment, is an authentication of the philosophy behind African bioethics that asserts that the experiences of suffering which the individual might go through are also communal experiences. The community is integral to the patient's decision‐making and healing processes.^106^

In the same vein of thought, Kasenene argues:

…one cannot regard even one's own life as purely personal property or concern. It is the group which is the owner of life… For that reason, one's health is a concern for the community, and a person is expected to preserve this life for the good of the group.^107^

Metz rejects that, according to Ubuntu, it is the group, which is the owner of life, and defends maintaining confidentiality based on Ubuntu grounds in general (taking into account some conditions): 108

… a medical (healthcare) professional should maintain confidentiality (unless there is serious risk to, say, the general public) when it comes either to a person's medical conditions that do not risk impairing his ability to live up to his special obligations or to third parties who are not the ones owed such special obligations. If sharing a way of life means interacting with others on a voluntary basis, then one may not divulge intimate information about them without their consent, supposing they are upholding their obligations to others.^109^

However, Metz mentions two exceptions to a confidentiality requirement in clinical practice. The first exception is based on patients with a duty of providing material aid to family members or those related to them. In such a situation, patients are obliged to inform their dependents of any foreseeable risks that might affect their ability to continue providing support. If a patient refuses to share this information, a healthcare professional would not be wrong to disclose these risks to the dependents.^110^ The second exception is a situation in which a genetic tester after conducting a test on a woman as a part of treatment discovers that the male partner incorrectly believes that he is the biological father of a child. Should the genetic tester disclose confidential information to the male partner? Generally, Ubuntu's moral perspective suggests that a tester should refrain from disclosing confidential information to prevent a family breakdown. However, according to Metz's interpretation of Ubuntu as a relational moral theory, the genetic tester would not be acting wrongly by disclosing confidential information to the male partner.^111^

Fulfilling these patients' confidential rights, as some authors have called them, remains morally challenging in a society that prizes communal relationships and strong familial relationships. An Ubuntu ethical framework would justify disclosing confidential patient information to family members *under some circumstances*. Some study participants expressed such a position, including situations where family members are involved in patient's wellbeing like paying medical costs. Under these circumstances, it would be challenging for these healthcare professionals not to disclose patient's health confidential information to the family members because the family and social networks play a significant role in the patient's healthcare decisions especially those involving medical costs. At the same time, the code of ethics and professional conduct developed by healthcare professional boards in Tanzania (Medical Council of Tanganyika and Tanzania Nursing and Midwifery Council) indicates confidentiality as one of the core principles on which a practitioner‐client relationship is built.^112^ For instance, the Medical Council of Tanganyika indicates confidential information can only be disclosed under these conditions, consent of a client is duly obtained, the disclosure is in compliance with the requirement of law, and the disclosure is in the interest of the public or community.^113^ However, in reality, as our study findings indicate, fulfilling this duty among healthcare professionals seems challenging due to the dominant communal values and familial relationships in our society. So, there is a need to find ways to deal with these inherent moral challenges of confidentiality and relational ethical viewpoints in the Tanzanian context. This can be done by integrating cultural value systems into medical ethics curricula or offering on‐the‐job training to healthcare professionals in order to try to balance autonomy‐based and communal relations perspectives in healthcare settings. Also, developing guidelines that suggest ways to address these moral challenges in clinical practice.

We recommend that CESS services could take into consideration the local contexts such as diversities in ethical frameworks in deliberating on moral challenges. Ubuntu ethics can provide one important framework when deliberating on moral challenges.^114^ Ubuntu ethics can be applied in ethics reflection in healthcare settings in various ways including aiding in the initial awareness and identification of the moral challenges occurring in the clinical setting, assisting in the analysis and argumentation of moral dilemmas and cases in the clinical setting. Also, Ubuntu ethics framework could assist CES services in situating an ethical issue within the cultural and social realm.

#### 4.3.3 Withdrawal of treatment

Participants highlighted that there are limited ethical guidelines and regulations to advice them on issues related to withdrawal of treatment, minimizing care or stopping aggressive medical care which is not beneficial to patients. The same finding is reported in a study among Ethiopian physicians.^115^ Yet, most of the participants confirmed to have standard operating procedures encompassing instructions and specific processes and protocols for carrying out their daily clinical tasks related to patient care protocols, infection control measures, medical administration procedures, emergency response protocols, and administrative tasks. It is suggested that ethical guidelines and regulations might assist healthcare professionals in decision‐making and maintaining professional standards.^116^ Our study found that most healthcare professionals lacked ethics support services that might aid them in resolving moral challenges related to withdrawal from treatment.

#### 4.3.4 Conflicts between professional judgment and religious and alternative treatment

Some participants reported challenges associated with refusal of treatment based on religious grounds and preference for alternative treatment. One dilemma concerned either aborting the fetus for the sake of saving the life of a pregnant woman or respecting the mother's and family's wishes to decline abortion, which might result in the death of both the woman and the fetus. In this situation, participants were ready to perform an abortion on the medical grounds, but the pregnant woman and the family members declined due to religious grounds. The Tanzanian abortion law as inscribed in the Penal Code and implied in the Country's constitution criminalizes illegal abortion as an offence against morality.^117^ Abortion is legally permitted by the Tanzanian legislation in defense of the health and life of a pregnant woman and states that in such circumstances a person is not criminally responsible for performing an abortion in good faith and with reasonable care and skills (Section 230 of the Penal Code of Tanzania, Cap. 16, Revised Edition, 2022).

Religious institutions in Tanzania primarily influence anti‐abortion views even on medical grounds through leaders and religious organizations.^118^ A Pew Forum survey indicates that 93% of Tanzanians think religion is essential in their lives, whereby Christianity (61%), and Islam (35%) are major religions in the country.^119^ Religious perspectives could be quite important for clinical ethics discussions, and healthcare professionals can discuss religious perspectives when challenging situations arise. Moral case deliberation models, such as the six‐step Centre for Medical Ethics model, offer a forum for ethics reflection.^120^ The model facilitates free and honest discussions on moral challenges by identifying key values, principles, laws, and regulations at stake. Therefore, it would offer an opportunity to incorporate religious considerations on abortion as a moral challenge in clinical practice. It would encourage the involvement of all those affected by the decision, and religious leaders could be part of the deliberation. Assembling those affected would help illuminate the values and principles at stake for the challenge deliberated on. Consequently, deliberation models can help clinicians regarding religious convictions conflicting with required healthcare services.

## 5 STUDY LIMITATIONS

This study has many strengths but also some limitations.^121^ The selection of the departments studied was based on discussions between the first author and several local healthcare professionals. We recognize that including other hospital departments and units might have yielded different results.

Another study limitation is that the first author who is from Tanzania, conducted the interviews, transcribed and translated data from Swahili to English, and produced the initial draft of the paper. Despite not being formally trained in medicine and health, the first author possesses an understanding of clinical ethics and the moral implications in clinical practices. This understanding, along with the first author's socio‐cultural background and knowledge influenced the data analysis. However, to promote different perspectives on the data and its presentation, co‐authors contributed to the analysis.

We interviewed only physicians and nurses. We might have not captured the perspectives of other groups of healthcare professionals working in hospital settings such as pharmacists, laboratory technicians, and social workers. In addition, we did not seek the perspectives of patients, family members, and community representatives about moral challenges encountered during their interactions with healthcare professionals. Yet, family relations play a significant role in healthcare delivery in Tanzanian settings. Therefore, we recommend future studies about clinical ethics issues to take into consideration the key needs of other stakeholders in healthcare delivery such as patients, family members, religious leaders and community representatives.

Finally, we did not include healthcare professionals from the lower healthcare settings such as regional hospitals, district hospitals, healthcare centres, and dispensaries. Therefore, we recommend that in future studies healthcare professionals from such healthcare settings are included.

## 6 CONCLUSIONS

Healthcare professionals in Tanzanian hospitals encounter a variety of moral challenges in clinical practice. The most common moral challenges are related to conflicts between principles of healthcare ethics and the socio‐cultural contexts in which healthcare professionals operate. For instance, in some cases, the family members play a greater role than the patient in deciding a treatment. In other instances, family members are involved in the handling of the patient's confidential information. In addition, the study findings show that the limitation of guidelines and regulations on the withdrawal or minimization of treatment that seems not to benefit ill patients tends to leave healthcare professionals and family members in a dilemma. Under such situations, healthcare professionals encounter a challenge while advising family members on the best course of action.

Despite these moral challenges, the study findings show that healthcare professionals received limited training in clinical ethics, as a consequence several reported a limited understanding of clinical ethics. Based on the findings, we suggest more ethics training to prepare healthcare professionals to address moral challenges. This can be done by doing the following ‐ introducing a clinical ethics course as part of continuous professional development for healthcare professionals, integrating clinical ethical issues that are context‐based in the healthcare professional education curriculum, as well as linking healthcare ethics/bioethics units/departments in the health and allied sciences institutes to hospitals. Experts in clinical ethics or bioethics could offer clinical ethical consultation to hospital departments through meetings.

## CONFLICT OF INTEREST STATEMENT

The authors have no financial, personal, academic or any other interest that could be considered as a potential conflict of interest in the subject matter of this manuscript.

## Supporting information

Supporting information.

Supporting information.

Supporting information.

## Footnotes

1 Portillo, A. B., García‐Caballero, R., de Asúa, D. R., & Ruíz‐Valdepeñas, B. H. (2021). Which are the Most Prevalent Ethical Conflicts for Spanish Internists? Revista Clínica Española (English Edition). 221(7): 393‐399.

2 Rasoal, D., Kihlgren, A., James, I., & Svantesson, M. (2016). What Healthcare Teams Find Ethically Difficult: Captured in 70 Moral Case Deliberations. Nursing Ethics. 23(8): 825‐837.

3 Winkler, E. C. (2005). The Ethics of Policy Writing: How Should Hospitals Deal with Moral Disagreement about Controversial Medical Practices? Journal of Medical Ethics. 31(10): 559‐566.

4 Portillo, et al., op. cit. note 1.

5 Hurst, S.A., Perrier, A., Pegoraro, R., Reiter‐Theil, S., Forde, R., Slowther, A.M., Garrett‐Mayer, E., & Danis, M. (2007). Ethical Difficulties in Clinical Practice: Experiences of European doctors. Journal of Medical Ethics. 33(1): 51‐57.

6 DuVal, G., Clarridge, B., Gensler, G., & Danis, M. (2004). A National Survey of U.S. Internists’ Experiences with Ethical Dilemmas and Ethics Consultation. Journal of General Internal Medicine. 19(3): 251‐258.

7 Morley, G., Ives, J., & Bradbury‐Jones, C. (2019). Moral Distress and Austerity: An Avoidable Ethical Challenge in Healthcare. Health Care Analysis. 27(3): 185‐201

8 Haahr, A., Norlyk, A., Martinsen, B., & Dreyer, P. (2020). Nurses Experiences of Ethical Dilemmas: A Review. Nursing Ethics. 27(1): 258‐272.

9 Ibid.

10 Eriksson, H., Andersson, G., Olsson, L., Milberg, A. & Friedrichsen, M. (2014). Ethical Dilemmas Around the Dying Patient with Stroke: A Qualitative Interview Study with Team Members on Stroke Units in Sweden. Journal of Neuroscience Nursing. 46(3): 162‐170.

11 Portillo, et al., op. cit. note 1.

12 Hurst, et al., op. cit. note 5.

13 Braunack‐Mayer, A.J. (2001). What Makes a Problem an Ethical Problem? An Empirical Perspective on the Nature of Ethical Problems in General Practice. Journal of Medical Ethics. 27(2): 98‐103.

14 DuVal, et al., op. cit. note 6.

15 Westra, A.E., Willems, D.L., & Smit, B.J. (2009). Communicating with Muslim Parents: “the Four Principles” are not as Culturally Neutral as Suggested. European Journal of Pediatrics. 168(11): 1383‐1387.

16 Portillo, et al., op. cit. note 1.

17 Metz, T. (2010). An African Theory of Bioethics: Reply to Macpherson and Macklin. Developing World Bioethics. 10(3): 158‐163.

18 Metz, T. (2018). A Bioethic of Communion: Beyond Care and the Four Principles with Regard to Reproduction. In M. Soniewick (Ed.), The Ethics of Reproductive Genetics: Between Utility, Principles, and Virtues (pp. 49–66). Cham: Springer.

19 Behrens, K.G. (2018). A Critique of the Principle of ‘Respect for Autonomy’: Grounded in African thought. Developing World Bioethics. 18(2): 126‐134.

20 Ibid.

21 Defaye, F.B., Desalegn, D., Danis, M., Hurst, S., Berhane, Y., Norheim, O.F., & Miljeteig, I. (2015). A Survey of Ethiopian Physicians’ Experiences of Bedside Rationing: Extensive Resource Scarcity, Tough Decisions and Adverse Consequences. BMC Health Services Research: 15(1): 1‐8.

22 Miljeteig, I., Defaye, F., Desalegn, D., & Danis, M. (2019). Clinical Ethics Dilemmas in a Low‐Income Setting ‐ a National Survey Among Physicians in Ethiopia. BMC Medical Ethics. 20(1): 1‐13.

23 Sippel, D., Marckmann, G., Ndzie Atangana, E., & Strech, D. (2015) Clinical Ethics in Gabon: The Spectrum of Clinical Ethical Issues Based on Findings from In‐Depth Interviews at Three Public Hospitals. PLoS ONE. 10(7): 1‐15.

24 Ewuoso, C. (2020). Addressing the Conflict between Partner Notification and Patient Confidentiality in Serodiscordant Relationships: How can Ubuntu Help? Developing World Bioethics. 20(2): 74‐85.

25 Defaye, et al., op. cit. note 21.

26 Moodley, K., Rennie, S., Behets, F., Obasa, A.E., Yemesi, R., Ravez, L., Kayembe, P., Makindu, D., Mwinga, A., & Jaoko, W. (2021). Allocation of Scarce Resources in Africa During COVID‐19: Utility and Justice for the Bottom of the Pyramid? Developing World Bioethics. 21(1): 36‐43.

27 Ewuoso, op. cit. note 24.

28 McLean, E., Desalegn, D.N., Blystad, A., & Miljeteig, I. (2019). When the Law Makes Doors Slightly Open: Ethical Dilemmas Among Abortion Service Providers in Addis Ababa, Ethiopia. BMC Medical Ethics. 20(1): 1‐10.

29 Ewnetu, D.B., Thorsen, V.C., Solbakk, J.H., & Magelssen, M. (2020). Still a Moral Dilemma: How Ethiopian Professionals Providing Abortion Come to Terms with Conflicting Norms and Demands. BMC Medical Ethics. 21(1): 1‐7.

30 Miljeteig, et al., op. cit. note 22.

31 Sippel, et al., op. cit. note 23.

32 Ibid.

33 Ewuoso, C., Fayemi, A.K., & Aramesh, K. (2021). Ubuntu Ethics and Moral Problems in Traditional Bone‐Healing. Journal of Global Health Reports. 5(1): 1‐9.

34 Ewuoso, C.O. (2018). Beneficial Coercion in Psychiatric Care: Insights from African Ethico‐Cultural System. Developing World Bioethics. 18(2): 91‐97.

35 Athanas, R., Gasto, F., & Renatha, S. (2020). Factors Influencing Truth‐Telling by Healthcare Providers to Terminally ill Cancer Patients at Ocean Road Cancer Institute in Dar‐es‐Salaam, Tanzania. South African Journal of Bioethics and Law. 13(2): 108‐113.

36 Kidayi, P.L., Manhica, H., Nakazzi, S., Mtuya, C.C., Aune, R.E., Serventi, F., Mahande, M.J., Björling, G. (2023). Caring for Breast Cancer Patients is a Work That Brings Sorrow: A Qualitative Interview Study with Nurses in Tanzania. SAGE Open Nursing. 9(1): 1‐10.

37 Vedasto, O., Morris, B., & Furia, F.F. (2021). Shared Decision‐Making Between Health Care Providers and Patients at a Tertiary Hospital Diabetic Clinic in Tanzania. BMC Health Services Research. 21(1): 1‐7.

38 Athanas, et al., op. cit. note 35.

39 Strong, A.E. (2017). Working in Scarcity: Effects on Social Interactions and Biomedical Care in a Tanzanian Hospital. Social Science & Medicine. 187(1): 217‐224.

40 Aboud, M., Bukini, D., Waddell, R., Peterson, L., Joseph, R., Morris, B.M., Shayo, J., Williams, K., Merz, J.F., & Ulrich, C.M. (2018). Do Tanzanian Hospitals Need Healthcare Ethics Committees? Report on the 2014 Dartmouth/Penn Research Ethics Training and Program Development for Tanzania (DPRET) Workshop. South African Journal of Bioethics and Law. 11(2): 75‐79.

41 Lechasseur, K., Caux, C., Dollé, S., & Legault, A. (2018). Ethical Competence: An Integrative Review. Nursing Ethics. 25(6): 694‐706.

42 Poikkeus, T., Numminen, O., Suhonen, R., & Leino‐Kilpi, H. (2014). A Mixed‐Method Systematic Review: Support for Ethical Competence of Nurses. Journal of Advanced Nursing. 70(2): 256‐271.

43 Vanlaere, L., & Gastmans, C. (2007). Ethics in Nursing Education: Learning to Reflect on Care Practices. Nursing Ethics. 14(6): 758‐766.

44 Andersson, H., Svensson, A., Frank, C., Rantala, A., Holmberg, M., & Bremer, A. (2022). Ethics Education to Support Ethical Competence Learning in Healthcare: An Integrative Systematic Review. BMC Medical Ethics. 23 (1), 29.

45 Poikkeus, et al., op. cit. note 42.

46 Moodley, K., Kabanda, S.M., Kleinsmidt, A., & Obasa, A.E. (2021). COVID‐19 Underscores the Important Role of Clinical Ethics Committees in Africa. BMC Medical Ethics. 22(1): 1‐9.

47 Molewijk, B., Verkerk, M., Milius, H. and Widdershoven, G. (2008). Implementing Moral Case Deliberation in a Psychiatric Hospital: Process and Outcome. Medicine, Health Care and Philosophy. 11(2): 43‐56.

48 Hem, M.H., Molewijk, B., Gjerberg, E., Lillemoen, L. and Pedersen, R. (2018). The Significance of Ethics Reflection Groups in Mental Health Care: A Focus Group Study Among Health Care Professionals. BMC Medical Ethics. 19(1): 1‐14.

49 Mohamed, A.M., Ghanem, M.A., & Kassem, A. (2012). Knowledge, Perceptions and Practices Towards Medical Ethics Among Physician Residents of University of Alexandria Hospitals, Egypt. Eastern Mediterranean Health Journal. 18(9): 935‐945.

50 Asare, P., Ansah, E.W., & Sambah, F. (2022). Ethics in Healthcare: Knowledge, Attitude and Practices of Nurses in the Cape Coast Metropolis of Ghana. PLoS One. 17(2): 1‐11.

51 O'Brien B.C., Harris, I.B., Beckman, T.J., Reed, D.A., & Cook, D.A. (2014). Standards for Reporting Qualitative Research: a Synthesis of Recommendations. Academic Medicine. 89(9): 1245‐1251.

52 Maluka, S., Chitama, D., Dungumaro, E., Masawe, C., Rao, K., & Shroff, Z. (2018). Contracting‐Out Primary Health Care Services in Tanzania Towards UHC: How Policy Processes and Context Influence Policy Design and Implementation. International Journal for Equity in Health. 17(1): 1‐13.

53 Ibid.

54 Braun, V., & Clarke, V. (2022). Thematic Analysis: A Practical Guide. Los Angeles: SAGE Publications.

55 Braun, V., & Clarke, V. (2006). Using Thematic Analysis in Psychology. Qualitative Research in Psychology. 3(2): 77‐101.

56 Braun, V., & Clarke, V. (2013). Successful Qualitative Research: A Practical Guide for Beginners. London: SAGE Publications.

57 Braun & Clarke, op. cit. note 55.

58 Braun & Clarke, op. cit. note 54.

59 Braun, V., & Clarke, V. (2024). Supporting Best Practice in Reflexive Thematic Analysis Reporting in Palliative Medicine: A Review of Published Research and Introduction to the Reflexive Thematic Analysis Reporting Guidelines (RTARG). Palliative Medicine. 38(6): 608‐16.

60 Miljeteig, I., Onarheim, K.H., Defaye, F.B., Desalegn, D., Norheim, O.F., Hevrøy, O., & Johansson, K.A. (2017). Ethics Capacity Building in Low‐Income Countries: Ethiopia as a Case Study. Tidsskriftet den Norske Laegeforening. 137(22): 1‐7.

61 Miljeteig, I., Berhane, F., & Desalegn, D. (2017). A Call for Open Access and Empathy Is Not Enough: Hands on Are Needed. American Journal of Bioethics. 17(10): 28‐30.

62 Andoh, C.T. (2011). Bioethics and the Challenges to its Growth in Africa. Open Journal of Philosophy. 1(2): 67‐75.

63 Ssebunnya, G.M. (2017). Beyond the Sterility of a Distinct African Bioethics: Addressing the Conceptual Bioethics Lag in Africa. Developing World Bioethics. 17(1): 22‐31.

64 Daily News Reporter. (2023, July 28). UDSM, Norway Agency to Address Ethical Dilemmas in Clinical Practices. Daily News. Retrieved October 8, 2024, from https://dailynews.co.tz/udsm-norway-agency-to-address-ethical-dilemmas-in-clinical-practices/.

65 Wambura, B. (2023, July 30). UDSM, UiO Tackle Ethical Dilemmas in Clinical Practice. The Citizen. Retrieved October 8, 2024, from https://www.thecitizen.co.tz/tanzania/news/national/udsm-uio-tackle-ethical-dilemma-in-clinical-practice-4320820.

66 Joseph, R., Kuhumba, S.K., & Kitula, L. (2021). Developing Bioethics Curriculum and Teaching Manual for Health Institutions in Tanzania: Needs assessment report. Unpublished Report funded by UNESCO – Tanzania.

67 Tiruneh, M.A., & Ayele, B.T. (2018). Practice of Code of Ethics and Associated Factors Among Medical Doctors in Addis Ababa, Ethiopia. PLoS One. 13(8): 1‐16.

68 Beauchamp, T., & Childress, J. (2019). Principles of Biomedical Ethics. (8th edition). New York: Oxford University Press.

69 Aboud, et al., op. cit. note 40.

70 Ibid.

71 Miljeteig, et al., op. cit, note 22.

72 DeBoer, R.J., Mutoniwase, E., Nguyen, C., Ho, A., Umutesi, G., Nkusi, E., Sebahungu, F., Van Loon, K., Shulman, L.N., & Shyirambere, C. (2021). Moral Distress and Resilience Associated with Cancer Care Priority Setting in a Resource‐Limited Context. The Oncologist, 26(7): 1189‐1196.

73 Miljeteig, et al., op. cit, note 22.

74 Fujii, T., Katayama, S., Miyazaki, K., Nashiki, H., Niitsu, T., Takei, T., Utsunomiya, A., Dodek, P., Hamric, A. and Nakayama, T. (2021). Translation and Validation of the Japanese Version of the Measure of Moral Distress for Healthcare Professionals. Health and Quality of Life Outcomes, 19(1): 1‐11.

75 Rushton, C.H., & Carse, A. (2016). Towards a New Narrative of Moral Distress: Realizing the Potential of Resilience. The Journal of Clinical Ethics. 27(3): 214‐218.

76 Young, P.D., & Rushton, C.H. (2017). A Concept Analysis of Moral Resilience. Nursing Outlook. 65(5), 579‐587.

77 Holtz, H., Heinze, K., & Rushton, C. (2018). Interprofessionals’ Definitions of Moral Resilience. Journal of Clinical Nursing. 27(3‐4): 488‐494.

78 DeBoer, et al., op. cit. note 72.

79 Morley, G., Sankary, L.R., & Horsburgh, C.C. (2022). Mitigating Moral Distress through Ethics Consultation. American Journal of Bioethics. 22(4): 61‐63.

80 Abbasi, S., Ghafari, S., Shahriari, M., & Shahgholian, N. (2019). Effect of Moral Empowerment Program on Moral Distress in Intensive Care Unit Nurses. Nursing Ethics. 26(5): 1494‐1504.

81 Rushton, C.H., Swoboda, S.M., Reller, N., Skarupski, K.A., Prizzi, M., Young, P.D., & Hanson, G.C. (2021). Mindful Ethical Practice and Resilience Academy: Equipping Nurses to Address Ethical Challenges. American Journal of Critical Care. 30(1): 1‐11.

82 Morley, G., & Horsburgh, C.C. (2023). Reflective Debriefs as a Response to Moral Distress: Two Case Study Examples. HEC Forum. 35(1): 1‐20.

83 Metselaar, S., & Molewijk, B. (2023). Fostering Moral Resilience Through Moral Case Deliberation. Nursing Ethics. 30(5), 730‐745.

84 United Republic of Tanzania. (2018). Current expenditure on health. Retrieved April 18, 2024, from https://knoema. com/atlas/United‐Republic‐of‐Tanzania.

85 Kagaigai, A., Anaeli, A., Grepperud, S., & Mori, A.T. (2023). Healthcare Utilization and Catastrophic Health Expenditure in Rural Tanzania: Does Voluntary Health Insurance Matter? BMC Public Health. 23(1): 1‐13.

86 Molewijk, B., Slowther, A., &Aulisio, M. (2022). Clinical Ethics: Support. In H. ten Have (Ed.), Encyclopedia of Global Bioethics (pp. 562 – 570). Cham: Springer International Publishing.

87 Ibid.

88 We thank the reviewer's constructive comment to include the roles of CES services at the institutional level, beyond addressing individual healthcare professionals’ cases of moral distress.

89 Magelssen, M., Karlsen, H., Pedersen, R., & Thoresen, L. (2020). Implementing Clinical Ethics Committees as a Complex Intervention: Presentation of a Feasibility Study in Community Care. BMC Medical Ethics. 21(1): 1‐9.

90 Geppert, C.M., & Shelton, W. (2016). Health Care Ethics Committees as Mediators of Social Values and the Culture of Medicine. AMA Journal of Ethics. 18(5): 534‐539.

91 Doran, E., Kerridge, I., Jordens, C., and Newson, A. J. (2016). Clinical Ethics Support in Contemporary Health Care: Origins, Practices and Evaluation. In E. Ferlie, K. Montgomery & A.R. Pedersen (Eds.), The Oxford Handbook of Health Care Management (pp. 164‐187). New York: Oxford University Press.

92 Magelssen, M., Miljeteig, I., Pedersen, R., & Førde, R. (2017). Roles and Responsibilities of Clinical Ethics Committees in Priority Setting. BMC Medical Ethics. 18(1): 1‐8.

93 Metz, T. (2010). African and Western Moral Theories in a Bioethical Context. Developing World Bioethics. 10(1): 49‐58.

94 Tangwa, G.B. (1996). Bioethics: An African Perspective. Bioethics. 10(3): 183‐200.

95 Jecker, N.S., & Atuire, C. (2022). Bioethics in Africa: A Contextually Enlightened Analysis of Three Cases. Developing World Bioethics. 22(2): 112‐122.

96 Metz, op. cit. note 93.

97 Behrens, K.G. (2013). Towards an Indigenous African Bioethics. South African Journal of Bioethics and Law. 6(1): 32‐35.

98 Metz, op. cit. note 93.

99 Metz, T. (2007). Toward an African Moral Theory. The Journal of Political Philosophy. 15(3): 321‐341.

100 Metz, T. (2011). Ubuntu as a Moral Theory and Human Rights in South Africa. African Human Rights Law Journal. 11(2): 532‐59.

101 Metz, T. (2010). Human Dignity, Capital Punishment, and an African Moral Theory: Toward a New Philosophy of Human Rights. Journal of Human Rights. 9(1): 81‐99.

102 Ibid.

103 We acknowledge the reviewer's substantial comments on clarifying privacy and confidentiality from the Ubuntu perspective.

104 Metz, T. (2022). A Relational Moral Theory: African Ethics in and Beyond the Continent. New York: Oxford University Press.

105 Ibid.

106 Murove, M.F. (2005). African Bioethics: An Explanatory Discourse. Journal for the Study of Religion. 18(1), 16‐36.

107 Kasenene, P. (2000). African Ethical Theory and the Four Principles. In R.M. Veatch (Ed.), Cross‐Cultural Perspectives in Medical Ethics (pp. 347–57). Toronto: Jones and Bartlett.

108 Metz, op. cit. note 104.

109 Ibid: 295.

110 Ibid.

111 Ibid.

112 See Medical Council of Tanganyika. (2005). Code of Ethics and Professional Conduct for Medical and Dental Practitioners in Tanzania; Tanzania Nursing and Midwifery Council. Retrieved October 8, 2024, from https://www.tnmc.go.tz/

113 Medical Council of Tanganyika. (2005). Code of Ethics and Professional Conduct for Medical and Dental Practitioners in Tanzania.

114 For further details about the role of ethical theory in ethical reflections, see Magelssen, M., Pedersen, R., & Førde, R. (2016). Four Roles of Ethical Theory in Clinical Ethics Consultation. American Journal of Bioethics, 16(9): 26‐33; and Porz, R., Landeweer, E., & Widdershoven, G. (2011). Theory and Practice of Clinical Ethics Support Services: Narrative and Hermeneutical Perspectives. Bioethics, 25(7): 354‐360.

115 Miljeteig, et al., op. cit. note 22.

116 Ibid.

117 United Republic of Tanzania. The Penal Code of Tanzania Cap.16, section 150(2022). Retrieved April 25, 2024, from http://www.un.org/depts/los/LEGISLATIONANDTREATIES/PDFFILES/TZA_penal_code.pdf.

118 Sambaiga, R., Haukanes, H., Moland, K. M., & Blystad, A. (2019). Health, Life and Rights: a Discourse Analysis of a Hybrid Abortion Regime in Tanzania. International Journal for Equity in Health. 18(1): 1‐12.

119 Pew Forum on Religion and Public Life. (2010). Tolerance and Tension: Islam and Christianity in sub‐Saharan Africa. Washington, DC: Pew Research Centre.

120 Førde, R., & Pedersen. R. (2011). Clinical Ethics Committees in Norway: What do they do, and does it Make a Difference? Cambridge Quarterly of Healthcare Ethics. 20(3): 389‐395.

121 We acknolwege the reviewer's comment to include the limitation section in this paper.

