# Supplementary material for: Moral challenges and understanding of clinical ethics in Tanzanian hospitals: Perspectives of healthcare professionals
Source: Dev World Bioeth. 2024 Oct 19;25(3):204–17. doi: 10.1111/dewb.12467 (PMC12407034; doi:10.1111/dewb.12467)
Supplement: Supplementary file 1 — Supporting information. [file DEWB-25-204-s003.docx]

**APPENDIX 1**

**GENDER, PROFESSION & SECTOR OF STUDY PARTICIPANTS**

| **CHARACTERISTIC** | **NO** | **FREQUENCY** |
| --- | --- | --- |
| **GENDER** | | |
| Female | 19 | 52% |
| Male | 17 | 48% |
| **PROFESSION** |  |  |
| Nurses | 21 | 58.3% |
| Physicians | 15 | 41.7% |
| **SECTOR** |  | |
| Government hospitals | 26 | 72.2% |
| Private owned hospital with government support | 10 | 27.8% |
