# Supplementary material for: Moral challenges and understanding of clinical ethics in Tanzanian hospitals: Perspectives of healthcare professionals
Source: Dev World Bioeth. 2024 Oct 19;25(3):204–17. doi: 10.1111/dewb.12467 (PMC12407034; doi:10.1111/dewb.12467)
Supplement: Supplementary file 2 — Supporting information. [file DEWB-25-204-s001.docx]

**APPENDIX 2**

***INDIVIDUAL INTERVIEW GUIDE FOR* HEALTHCARE PROFESSIONALS (NURSES & PHYSICIANS) IN THE HOSPITAL SETTINGS**

| **PART I: INTRODUCTION & DEMOGRAPHIC INFORMATION** |
| --- |
| Name of the Institution: |
| Date: |
| Gender: |
| Years of experience in healthcare: |
| Department: |
| Years working in the current department: |
| Position: |
| Area of specialisation: |
| Cadre: Nurse ( ) Physician ( ); Specialist ( ); Resident ( ); other – please specify |
| Leadership position: |
| Number of employees under your responsibility: |

**PART I: INTRODUCTORY QUESTIONS**

1. Have you got any kind of medical/clinical ethics education during your professional training?
2. If yes, to the above question, what kind of training?
3. How many hours or days for the training?
4. Did you ever hear about the term ethics? If so, could you describe to me how you would describe ethics?
5. How do you understand ethics/ clinical ethics in healthcare?
6. Do you and your colleagues use the term ‘ethics’, now and then? If so, when, and in which kind of cases?
7. What are important values in your daily work (Values are the things that are important to us, what we consider to be good. (Context-based).

**PART III: QUESTIONS RELATED TO MORAL CHALLENGES IN THE HEALTHCARE SETTING**

1. Could you please describe one morally challenging situation you have ever experienced in your clinical practices? **(Probe questions)** What was the situation? And what made this morally challenging for you?
2. How did you experience facing this situation?
3. What did you do in order to cope with this situation?
4. What are common clinical moral challenges in your department? How are they handled?

**PART IV: QUESTIONS RELATED TO AVAILABLE MECHANISMS TO HANDLE MORAL CHALLENGES IN THE HEALTHCARE SETTINGS**

1. How do you usually deal with moral challenges in your department? How useful is the mechanism used to handle moral challenges in your department?
2. How should morally challenging situations be dealt with? What can be done to support healthcare personnel facing moral challenges?
3. What are potential challenges in dealing with moral challenges in your workplace?

**PART V: QUESTIONS RELATED TO CLINICAL ETHICS COMMITTEE**

1. Do you know about the clinical ethics committee, or have you ever experienced one before? A CEC has three key functions – i) ethical deliberations in the healthcare setting, ii) policy advisory role, and iii) educational task. A CEC provides inter-professional advice – not control/sanction/judicial or disciplinary committee.
2. What facilitators (resources) are available in establishing a clinical ethics committee?
3. What could be barriers that might hinder an establishment of a clinical ethics committee or the effective functioning of a clinical ethics committee in your hospital?
4. In your opinion, what should be done in addressing morally challenging situations in the hospital setting?
