# Supplementary material for: Moral challenges and understanding of clinical ethics in Tanzanian hospitals: Perspectives of healthcare professionals
Source: Dev World Bioeth. 2024 Oct 19;25(3):204–17. doi: 10.1111/dewb.12467 (PMC12407034; doi:10.1111/dewb.12467)
Supplement: Supplementary file 3 — Supporting information. [file DEWB-25-204-s002.docx]

**APPENDIX 3**

**THEMES, SUB-THEMES, CODES AND ILLUSTRATIVE QUOTES**

| **Themes** | **Sub-themes** | **Codes** | Illustrative quotes |
| --- | --- | --- | --- |
| **Ethics education in healthcare** | - Avenues of ethics and medical ethics education – formal education, online training, and workshops  - Learned about codes and conduct guiding clinical practices.  - Studied more about medical scientific aspects | - Professionalism  - Patient-doctor relationship  - Principles  - Dilemmas  - Decision-making  - Medical science  - No trained ethicists  - Ethics  - Medical Experts  - Online study  - Healthcare services  - Customer challenges  - Medical ethics  - Healthcare code of conduct | I have been exposed to medical ethics mostly through my initiative to study online (#15, male physician, hospital A). |
|  |  |  | We were taught how to work efficiently and address customer challenges when providing healthcare services…. (#20, male nurse, hospital B). |
|  |  |  | I have … received some training on …. the healthcare code of conduct with ethical components …. (#10, male physician, hospital A). |
|  |  |  | … the focus was on professionalism and the patient-doctor relationship, but the actual ethical aspects, such as principles, dilemmas, and decision-making, were only touched upon very briefly…. Unfortunately, when we were studying, no trained ethicists were available, so experts from the medical profession often taught us. They put more focus on the medical science aspects rather than ethics. (#14, female physician, hospital A). |
| **Understanding of ethics and clinical ethics** | - Reference to bioethical principles | - Patient’s rights  - Harm  - Respect  - Beneficial to the patients  - Confidentiality  - Patient’s information | Patient's rights are upheld without causing harm (#11, female physician, hospital A). |
|  |  |  | Doing something beneficial for the patient and maintaining the confidentiality of the information provided by the patient (#6, male physician, hospital A). |
|  |  |  | … are provided, the reception and management of patient or client information” (#25, male nurse, hospital B). |
|  |  |  |  |
|  | - Adherence to the codes of conduct, regulations, guidelines and procedures | - Laws  - Code of conduct  - Code of conduct for doctors and nurses | Meeting their needs and offering quality services (#32, female nurse, hospital C) |
|  |  |  | Following regulations, guidelines, and procedures” (#33, male physician, hospital C) |
|  |  |  | …. it could mean rules established to guide healthcare professionals in providing healthcare services… (#1, male nurse, hospital A). |
|  |  |  | Norms that are essential in the clinical practice related to communication because kindness and cheerfulness could be given to patients through it (#13, male physician, hospital A). |
|  |  |  | Values such as honesty and exhibiting good customer care to the patients in the clinical practice were also associated with clinical ethics (#23, female nurse, hospital B). |
| **Moral challenges in clinical practice** | - **Decision-making and communication in the clinical practice** | - Decision-making  - Family  - Family members  - Relatives  - Parents  - Withdraw of curative treatment  - Friends  - Neighbours  - Communal approach  - Patients  - Confidential information  - Counselling  - Inability to afford medical costs  - Communal approach  - Organ transplantation  - Kidney donation  - Communication  - Truth-telling | In our setting, very often an entire family is significant in deciding a patient's treatment because we have a communal approach. (#9, female nurse, hospital A). |
|  |  |  | Patients don't make decisions for themselves. Even their confidential information is handled by their family members, friends, or neighbours…. (#9, female nurse, hospital A). |
|  |  |  | We had a patient in the ICU who was on maximum life support, and the relatives came and requested to take the patient home because they couldn't afford to care for them here…. (#34, female physician, hospital C). |
|  |  |  | …a sick child who needed close medical attention and care from a doctor, but the parents wanted the child to be taken off oxygen and taken home. …. they went home against medical advice … it seems the mother herself wasn't ready to see her baby discharged against medical advice. Even though her decisions might differ from the husband's, he is still the one with the means and the provider of finances for medical treatment(#11, female physician, hospital A). |
|  |  |  | A patient undergoes a procedure, for example, intubated, and is on a ventilation machine. There is no process for them to be part of the decision-making, even though they are informed about the situation. (#9, female nurse, hospital A). |
|  |  |  | We advised them to deliver the baby quickly because the mother wouldn't recover because she was seven months pregnant, the baby could have survived because babies born at that stage can survive. But there were discussions about what would happen if the baby was delivered prematurely… They presented their arguments, but the challenge was not resolved… eventually, the pregnant woman's heart stopped before delivering the baby. (#25, male nurse, hospital B). |
|  |  |  | …a young man had decided to donate a kidney to his uncle, but in front of people, his mother had accepted, and then later, when they were alone, she told him not to come back home if he donates his kidney. So, I was in a difficult situation to advise a potential donor whether to donate the kidney or accept the ' 'mother's position. (#14, female physician, hospital A). |
|  |  |  | For example, there are moments when a patient comes with severe illness, but you find they are having brain death. Do you remove them from the machine? How do you tell the parents or family members? How do you convince them when they see the heartbeat of their patient, yet in the actual sense, it is the machine helping the patient to breathe? (#15, male physician, hospital A). |
|  |  |  | Although the main challenge is that legally we still don’t have a guideline about either minimizing or stopping treatment for terminally ill patients. I am in the process of having empirical evidence to write a policy paper to inform the Ministry of Health about the need to establish legal and ethical guidelines that will assist healthcare practitioners in making complex decisions. (#15, male physician, hospital A). |
|  |  |  | One of the challenges that often arises is when dealing with patients who have reached a condition where they can no longer recover, for example, when specific organs have failed to function, and the brain is also unable to function. There are no guidelines to aid you in how to handle such a situation. However, keeping them on life support machines increases the medical costs for their families, who sometimes, after understanding the patient's condition, request to have them removed from life support to reduce the financial burden. (#19, male nurse, hospital B). |
|  | **Scarcity of medical resources and prioritization in clinical practice** | - Multiple disabilities  - Health conditions  - Limited ventilation machines  - Law prohibitions  - Scarcity of blood  - Insufficient protective equipment | … there are situations where no matter what we do, a child cannot recover because they were born with multiple disabilities or … their brain has been damaged due to lack of oxygen. Babies under such health conditions require full-time ventilation for their survival. But the ventilation machines are limited. Then another child with better conditions comes along who needs a ventilation machine. Now you are faced with a dilemma: the law prohibits taking off the machine a baby with multiple disabilities. Even if the child survives, his quality of life may not be good. So, do you turn off the machine, or wait until the terminally ill child dies? (#1, male nurse, hospital A). |
|  |  |  | I faced a case where there were three patients needing blood, but the laboratory only had one unit of blood from a group O donor, which could be given to only one of them. (#26, female physician, hospital B). |
|  |  |  | There was insufficient protective equipment, so there were moments when you would see a patient suffering but couldn't get close. (#25, male nurse, hospital B). |
|  | **Withdrawal of treatment** | - Natural death  - Tanzanian guidelines  - Terminally ill patient  - Saving the life of a patient  - Life support  - Medical costs  - Family  - Patient’s conditions  - Request to remove from life support machine  - Financial burden | According to guidelines, you can't remove them from life support machines until they die naturally. However, keeping them on life support increases the medical costs for their families, who sometimes, after understanding the patient's condition, request to have them removed from life support to reduce the financial burden. (#19, male nurse, hospital B). |
|  |  |  | … when you have a patient who won't recover and is on oxygen, and then another patient needs a machine to save their life potentially. However, Tanzanian guidelines don't allow us to remove the ventilation machine from the terminally ill patient, even if it could save the life of a patient with a high chance of recovering. (#18, female physician, hospital B).  they may require dialysis, which is very costly, and at the same time, the patient's condition may have deteriorated significantly… (#19, male nurse, hospital B). |
|  | **Conflicts between professional judgment, religious beliefs and alternative treatments** | - Termination of pregnancy  - Family and relatives’ belief in God’s miracle  - Sickle cell disease  - Family members  - Refusal of surgery  - alternative treatment  - Spiritual therapies | I have faced the challenge of a pregnant woman with a serious health issue that required the termination of her pregnancy to save her life. Given her condition and the stage of her pregnancy, termination was necessary. However, her family and relatives believed that God would perform a miracle, so they insisted on keeping the pregnancy. (#20, male nurse hospital B). |
|  |  |  | a young boy, around 8 years old … His problem was sickle cell disease, and his blood level was deficient. … They told us that if we gave him blood, we would be sued…. (#36, male nurse, hospital C). |
|  |  |  | It was a young person who had been in an accident and got a fracture on one of his legs. He also had an open wound that was bleeding. Their family members refused to allow him to undergo surgery because they preferred alternative treatment (traditional medicine)…. (#28, female nurse, hospital C). |
|  |  |  | … the patient left the hospital premises and went for spiritual therapies – prayers, but unfortunately, the patient died. (#12, male nurse, hospital A) |
